# Comparison of Three Commercially Available Dengue NS1 Antigen Capture Assays for Acute Diagnosis of Dengue in Brazil

**DOI:** 10.1371/journal.pntd.0000738

**Published:** 2010-07-06

**Authors:** Monique da Rocha Queiroz Lima, Rita Maria Ribeiro Nogueira, Hermann Gonçalves Schatzmayr, Flavia Barreto dos Santos

**Affiliations:** Flavivirus Laboratory, Oswaldo Cruz Institute/FIOCRUZ, Rio de Janeiro, Brazil; Universidade de São Paulo, Brazil

## Abstract

**Background:**

Dengue is associated with explosive urban epidemics and has become a major public health problem in many tropical developing countries, including Brazil. The laboratory diagnosis of dengue can be carried out using several approaches, however sensitive and specific assays useful to diagnose in the early stage of fever are desirable. The flavivirus non-structural protein NS1, a highly conserved and secreted glycoprotein, is a candidate protein for rapid diagnosis of dengue in endemic countries.

**Methodology/Principal Findings:**

We aimed to evaluate the potential use of 3 commercial kits in a panel of 450 serum samples for early diagnosis of dengue in Brazil. The PanBio Early ELISA (PanBio Diagnostics) showed a sensitivity of 72.3% (159/220) and a specificity of 100%, while the sensitivity of the Platelia™ NS1 assay (Biorad Laboratories) was 83.6% (184/220). However, the highest sensitivity (89.6%; 197/220) was obtained by using the NS1 Ag Strip (Biorad Laboratories). A lower sensitivity was observed in DENV-3 cases by all 3 kits. Serum positive by virus isolation were more often positive than cases positive by RT-PCR by all three assays and a higher detection rate was observed during the first four days after the onset of the symptoms. The presence or absence of IgM showed no influence in the confirmation by the pan-E Early ELISA (*P* = 0,6159). However, a higher confirmation by both Platelia™ NS1 (Biorad) and Dengue NS1 Ag Strip (Biorad) in the absence of IgM was statistically significant (*P*<0,0001 and *P* = 0,0008, respectively). Only the Platelia™ NS1 test showed a higher sensitivity in confirming primary infections than secondary ones.

**Conclusions/Significance:**

The results indicate that commercial kits of dengue NS1 antigen are useful for the laboratory diagnosis of acute primary and secondary dengue. It can be used in combination with the MAC-ELISA for case detection and as screening test to complement viral isolation.

## Author Summary

Dengue is the one of the most prevalent arthropod-borne viral diseases in tropical regions of the world. Manifestations may vary from asymptomatic to potentially fatal complications. Laboratorial diagnosis is essential to diagnose dengue and differentiate it from other diseases. Dengue virus non-structural protein 1 (NS1) may be used as a marker of acute dengue virus infection. Our results, based in the comparison of three NS1 antigen capture assays available, have shown that this approach is reliable for the early diagnosis of dengue infections, especially in the first four days after the onset of the symptoms. A lower sensitivity was observed in DENV-3 cases. Serum positive by virus isolation were more often detected than those positive by RT-PCR by all three assays. Only the Platelia™ NS1 test showed a higher sensitivity in confirming primary infections than secondary ones. In conclusion, NS1 antigen capture commercial kits are useful for diagnosis of acute primary and secondary dengue infections and, in endemic countries where secondary infections are expected to occur, may be used in combination with MAC-ELISA to increase the overall sensitivity of both tests.

## Introduction

Dengue is associated with explosive urban epidemics and has become a major public health problem [1]. Annually, the World Health Organization estimates that 50–100 million people are infected with dengue virus (DENV) worldwide with estimated 250,000–500,000 cases of dengue haemorrhagic fever (DHF) and dengue shock syndrome (DSS) with about 25,000 deaths occurring. One or more of four serotypes of DENV (DENV1–4), a mosquito-borne, positive-strand RNA virus in the genus *Flavivirus*, family *Flaviviridae* cause the disease in more than 100 endemic countries in tropical areas [2].

The geographical spread of all four DENV serotypes throughout the subtropical regions of the world has led to larger and more severe outbreaks and the accurate and efficient diagnosis of the disease is important for clinical care, surveillance, pathogenesis studies and vaccine research. Furthermore, an efficient diagnosis is an important tool to support Epidemiological Surveillance Programs considering the difficulties in confirming dengue cases based only on the clinical symptoms, especially during inter-epidemic periods.

Dengue is an enveloped virus with a single-stranded, positive sense RNA genome of about 11 kb containing a single open reading frame enconding a single polyprotein co- and pos-translationally cleaved into 3 structural (C, prM and E) and 7 nonstructural proteins (NS1, NS2A, NS2B, NS, NS4A, NS4B and NS5) [3].

Dengue is a major public health problem in many tropical and subtropical countries in the world. The accurate and efficient diagnosis of dengue is important for clinical care, surveillance, pathogenesis studies, and vaccine research.

The most used techniques use for dengue serodiagnosis are based on the anti-DENV IgM and IgG detection by using MAC-ELISA and IgG-ELISA [4]. However, one of the limitations consists in the variations on the detection rate during the acute phase of the disease. Usually, it takes from 3 to 5 days after the onset of the symptoms to detect anti-DENV IgM and from 1 to 14 days to anti-DENV IgG to become detectable, depending on whether the patient has primary or secondary infections [5].

During the acute phase, however, the NS1 exists as secreted as well as a membrane-associated protein and both forms are demonstrated to be immunogenic [6], [7], [8], [9], [10]. High NS1 level was demonstrated to circulate in the acute phase of dengue by antigen capture ELISAs, found in the sera of patients with primary and secondary DENV infections, up to the ninth day after the onset of the symptoms [10], [11].

The availability of commercial kits for the detection of anti-DENV NS1 in acute serum provides an alternative to the existing methods such as PCR, serology and virus isolation. Previous studies have shown the sensitivity and specificity of NS1 capture commercial kits for the laboratorial diagnosis of dengue infections [12], [13], [14], [15], [16], [17], [18], [19].

Recently, the Brazilian Ministry of Health has establish this new approach in sentinel clinics throughout the country after the 2008 dengue epidemic, however without a full evaluation of the commercial tests available. In the study, we aimed to evaluate the sensitivity and specificity of 3 commercially-available dengue NS1 antigen kits to demonstrate its potential use for the early laboratory confirmation of acute dengue infection in Brazil. This constitutes the first report of a comparison of NS1 antigen capture assays performed in the country.

## Materials and Methods

### Ethics statement

The samples belong to a previously-gathered collection from an ongoing Project in the Laboratory approved by the Ethics Committee on Human Research (CEP: 274/05).

### Dengue cases and non- dengue cases definitions

Laboratory-positive DENV infection was defined in patients experiencing a febrile illness consistent with dengue according to WHO criteria [20] in which infection was confirmed by DENV isolation [21], detection of DENV RNA by RT-PCR [22], detection of anti-DENV IgM antibodies by MAC-ELISA[23], and/or a >4-fold rise in anti-DENV IgG-ELISA titer in paired acute and convalescent sera [24]. Individuals negative for DENV infection by using all the methods described above and health individuals were classified as non-dengue cases.

### Clinical samples

The serum samples (days 1^st^ to 9^th^ after the onset of the symptoms) analyzed in this study by the pan-E Early ELISA (PanBio Diagnostics, Brisbane, Australia- first generation), Platelia™ (Biorad Laboratories, Marnes-La-Coquette, France) and NS1 Ag Strip (Biorad Laboratories, Marnes-La-Coquette, France) belong to a previously-gathered serum collection of the Laboratory of Flavivirus at Oswaldo Cruz Institute, FIOCRUZ, Brazil, from epidemics occurred from 1986 to 2008. A panel of 450 sera (220 dengue positive sera and 230 non-dengue sera) was divided into eleven Groups as follows: Groups A to C, sera from patients infected with DENV-1 (*n* = 50), DENV-2 (*n* = 50), and DENV-3 (*n* = 58), respectively; Group D, sera from patients with dengue infection serologically confirmed by MAC-ELISA with negative virus isolation and RT-PCR (*n* = 62); Group E, sera from healthy individuals (*n* = 30); Group F, sera from individuals negative for dengue (*n* = 86); Group G, sera from yellow fever positive individuals (*n* = 20); Group H, sera from individuals vaccinated for yellow fever and negative for anti-DENV antibodies (*n* = 44); Group I, sera from measles patients (*n* = 16) and Group J, sera from rubella patients (*n* = 34).

### Dengue virus isolation

Virus isolation was performed by inoculation into C6/36 *Aedes albopictus* cell line [21] and isolates were identified by indirect fluorescent antibody test (IFAT) using serotype-specific monoclonal antibodies [25].

### Reverse transcriptase (RT) –PCR

RT—PCR for detecting and typing DENV was performed as described previously [22]. Briefly, consensus primers were used to anneal to any of the four DENV types and amplify a 511-bp product in a reverse transcriptase-polymerase reaction. A cDNA copy of a portion of the viral genome was produced in a reverse transcriptase reaction. After a second round of amplification (nested PCR) with type-specific primers, DNA products of unique sizes for each DENV serotype were generated and analyzed by gel electrophoresis.

### Immunoglobulin M (IgM) antibody capture ELISA (MAC-ELISA)

The *in-house* MAC-ELISA was carried out for dengue cases confirmation as described previously [23].

### Immunoglobulin G (IgG) antibody detection ELISA (IgG—ELISA)

The IgG—ELISA previously described by Miagostovich [24] was performed for the characterization of dengue immune response as primary or secondary infections in dengue cases previously confirmed by virus isolation, RT—PCR and/or MAC-ELISA. Briefly, 96-well plates were coated with hyper immune ascitic fluid (a mixture of anti-DENV-1 to 4), followed by the addition of a mixture of the four DENV antigens. Serum diluted 1∶40 was added to the first well and four-fold dilutions were carried out up to the eighth well. After incubation, anti-human IgG conjugated to horseradish peroxidase was added Acute phase serum samples (<6 days after onset of symptoms) with IgG-ELISA titers of 1∶160 or greater are considered to be secondary infections. Likewise, samples with titers >1∶10, 240 on days 6–9, or >1∶40, 960 on days 10–15 after onset are secondary responses.

### NS1 antigen capture assays

#### pan-E DENGUE EARLY ELISA (PanBio Diagnostics, Brisbane, Australia)

This first generation test is based on a one-step sandwich format microplate enzyme immunoassay to detect DENV NS1 antigen in human sera. The test uses horseradish peroxidase conjugated anti-NS1 monoclonal antibody with preservative. If NS1 antigen is present in the sample, an immune-complex MAb-NS1-MAb/peroxidase will be formed. The acute serum specimens were allowed to thaw to laboratory ambient temperature (21–22°C). Briefly, 100 µL diluted test samples and controls were pipetted into their respective microwells and incubated for 60 min at 37°C. After a six-time washing steps, 100 µL HRP conjugate anti-NS1 MAb were pipetted into each well and plate was incubated for 60 min at 37°C. After a six-times washing step, 100 µL of substrate was added into each well and incubated for 10 min at room temperature in the dark. The presence of immune-complex was demonstrated by a color development and the enzymatic reaction was stopped by adding 100 µL 1M H_3_PO_4_. The optical density (OD) reading was taken with a spectrophotometer at a wavelength of 450–620 nm and the amount of NS1 antigen present in an individual serum sample was determined by comparing the OD of the sample to the OD of the cut-off control serum. Results were calculated as “Panbio units” with results <9.0, 9.0–11.0, and ≥11.0 defined as negative, inconclusive, and positive, respectively. Inconclusive samples were re-tested to confirm the result.

#### Platelia™ Dengue NS1 Ag-ELISA (Biorad Laboratories, Marnes-La-Coquette, France)

The test is based on a one-step sandwich format microplate enzyme immunoassay to detect DENV NS1 antigen in human serum or plasma. The assay uses murine monoclonal antibody for capture and revelation. If NS1 antigen is present in the sample, an immune-complex MAb-NS1-MAb/peroxidase will be formed. Briefly, the acute serum specimens were allowed to thaw to laboratory ambient temperature (21–22°C). Sample diluent (50µl), respective samples and controls (50 µl each) and 100 µl of diluted conjugate were incubated for 90 min at 37°C within the respective microplate wells coated with purified mouse anti-NS1 monospecific antibodies. After a six-times washing step, 160µl of substrate was added into each well and incubated for 30 min at room temperature in the dark. The presence of immune-complex was demonstrated by a color development and the enzymatic reaction was stopped by adding 100µl 1N H_2_SO_4_. The optical density (OD) reading was taken with a spectrophotometer at a wavelength of 450–620 nm and the amount of NS1 antigen present in an individual serum sample was determined by comparing the OD of the sample to the OD of the cut-off control serum.

#### Dengue NS1 Ag STRIP (Biorad Laboratories, Marnes-La-Coquette, France)

Dengue NS1 Ag STRIP is an immunochromatographic test (ICT) for the rapid detection of NS1 antigen. Briefly, one drop of migration buffer was added to 50 µL serum in a specimen tube and a strip was placed in the tube. The strip has two lines: a control line (C) (‘biotin – gold colloidal particles coated with streptavidin’ complex) and a test line (T) (‘monoclonal anti-NS1 antibodies (mAb) – NS1 Ag – gold colloidal particles coated with anti-NS1 mAb’ complex). The appearance of the T and C lines after a migration time of 15 minutes (min) indicates a positive result. The appearance of the C line alone indicates a negative result. If the C line is not present, the test is considered invalid and is repeated. It is recommended that strips giving ambiguous (faint color at the T line) or negative results are put back in the tube after the initial reading and left for a further 15 min for re-evaluation. We evaluated all samples at 15 min (ICT 15 min) and then at 30 min (ICT 30 min).

### Data and statistical analysis

The sensitivities, specificities, efficiency, negative and positive predicted values were calculated as follows:

Sensitivity: a/a+c X 100%

Specificity: d/d+b X 100%

Efficiency: a+d/a+b+c+d X 100%

Negative Predicted Value: d/d+c X100%

Positive Predicted Value: a/a+b X 100%; where: a = number of true positive, b = number of false positive, c = number of false negative and d = number of true negative.

The derived data was tabulated in appropriate worksheets using the Microsoft Excel and evaluated by chi-square test using the Epi Info 6 (Center for Disease Control and Prevention, Atlanta) for any statistical significant association.

## Results

### Overall sensitivities of the NS1 antigen capture tests

A panel of 450 (*n* = 220 dengue cases and *n* = 230 non-dengue cases) was used to evaluate three NS1 antigen capture tests commercially available. The overall sensitivities were 72.3% (159/220) for the pan-E Early ELISA (PanBio) test, 83.6% (184/220) for the Platelia™ NS1 (BioRad) kit, and 89.6% (197/220) for the NS1 Ag Strip kit (BioRad), Table 1. The differences observed in the sensitivities between the three kits analyzed were statistically significant (*P* = 0.0009).

**Table 1:** Sensitivity and specificity of 3 commercially available kits for NS1 antigen capture in dengue sera and controls.

	NS1 antigen capture kitNo. of sera with indicated result/total no. tested^b^					
Groups^a^	pan-E DENGUE EARLY ELISA		Platelia™ Dengue NS1 Ag-ELISA		Dengue NS1 Ag STRIP	
	Negative	Positive	Negative	Positive	Negative	Positive
A (DENV1 cases; *n* = 50)	13/50 (26.0)	37/50 (74.0)	1/50 (2.0)	49/50 (98.0)	1/50 (2.0)	49/50 (98.0)
B (DENV2 cases; *n* = 50)	9/50 (18.0)	41/50 (82.0)	5/50 (10.0)	45/50 (90.0)	1/50 (2.0)	49/50 (98.0)
C (DENV3 cases; *n* = 58)	20/58 (34.5)	38/58 (65.5)	8/58 (13.8)	50/58 (86.2)	7/58 (12.0)	51/58 (88.0)
D (IgM positive cases; *n* = 62)	19/62 (31.0)	43/62 (69.0)	22/62 (35.5)	40/62 (64.5)	14/62 (22.6)	48/62 (77.4)
**Total of Groups A–D**	61/220 (27.7)	159/220 (72.3)	36/220 (16.4)	184/220 (83.6)	23/220 (10.5)	197/220 (89.5)
E (healthy individuals; *n* = 30)	30/30 (100)	0/30	30/30 (100)	0/30	30/30 (100)	0/30
F (individuals negative for dengue; *n* = 86)	86/86 (100)	0/86	85/86 (98.8)	1/86	86/86 (100)	0/86
G (yellow fever positive cases; *n* = 20)	20/20 (100)	0/20	20/20 (100)	0/20	20/20 (100)	0/20
H (individuals vaccinated for yellow fever; *n* = 44)	44/44 (100)	0/44	43/44 (97.7)	1/44	43/44 (97.7)	1/44
I (measles cases; *n* = 16)	16/16 (100)	0/16	16/16 (100)	0/16	16/16 (100)	0/16
J (rubella cases; *n* = 34)	34/34 (100)	0/34	33/34 (97.0)	1/34	33/34 (97.0)	1/34
**Total of Groups E–J**	230/230 (100)	0/230	227/230 (98.7)	3/230	228/230 (99.1)	2/230

a Subjects in groups A to D had confirmed DENV infection; subjects in groups E to J had no DENV infection.

b Values in parentheses are percentages.

### NS1 sensitivities in relation to viral serotype

The pan-E Early ELISA (PanBio) showed a higher sensitivity in confirming DENV-2 infections (Group B; 82.0%) than confirming DENV-1 and DENV-3 infections. The Platelia™ NS1 kit (BioRad) was more sensitive in the detection of DENV-1 cases (Group A; 98.0%) than in the detection of DENV-2 and DENV-3 infections. The Dengue NS1 Ag Strip kit (BioRad) showed the same sensitivity in confirming DENV-1 and DENV-2 infections (98.0%). DENV-3 infections were the detected less often by all the three kits tested (65.5%, 86.2% and 88.0% for the pan-E Early ELISA (PanBio), the Platelia™ NS1 kit (BioRad) and the Dengue NS1 Ag Strip kit (BioRad), respectively (Table 1).

### NS1 specificities and cross-reactivity in patients with other confirmed diagnoses and yellow fever vaccination

Specificities were 100%, 98.7% and 99.1% for the PanBio kit, for the Platelia™ NS1 kit (Biorad) and for the NS1 Ag Strip (BioRad), respectively, based on the analysis of sera of healthy individuals (Group E) and individuals negative for dengue (Group F), Table 1. No cross-reactivity was observed with sera from yellow fever infected patients (Group G); however both Biorad kits showed cross-reactivity with one yellow fever vaccinee (Group H). None of the measles sera (Group I) were recognized by the NS1 tests. One rubella positive case (Group J) showed cross-reactivity with both Platelia™ NS1 kit (Biorad) and for the NS1 Ag Strip (BioRad) kit. The overall evaluations according to the different Groups analyzed are shown in Table 1.

### NS1 sensitivities according to the different acute diagnosis methods

A higher sensitivity (71.5%, 94.8% and 98.7%) was observed in cases positive by virus isolation only than in cases previously positive by RT-PCR (62.3%, 82.3% and 82.3%) for the pan-E Early ELISA, Platelia™ NS1 and Dengue NS1 Ag Strip, respectively (Table 2).

**Table 2:** Sensitivities of 3 NS1 antigen capture assays in patients with clinical diagnosis of acute dengue confirmed by RT-PCR and/or virus isolation and by MAC-ELISA.

	NS1 antigen capture kitsNo. of sera with indicated result/total no. tested^a^		
Dengue case confirmation	pan-E DENGUE EARLY ELISA	Platelia™ Dengue NS1 Ag-ELISA	Dengue NS1 Ag STRIP
RT-PCR only (*n* = 45)	28/45 (62.3)	37/45 (82.3)	37/45 (82.3)
Virus isolation only (*n* = 77)	55/77 (71.5)	73/77 (94.8)	76/77 (98.7)
RT-PCR and virus isolation (*n* = 36)	33/36 (91.7)	34/36 (94.5)	36/36 (100)
MAC-ELISA only	43/62 (69.4)	40/62 (64.5)	48/62 (77.4)
Total	159/220 (72.3)	184/220 (83.6)	197/220 (89.6)

a Values in parentheses are percentages.

### NS1 sensitivities in the presence and absence of IgM

The detection rate by the pan-E Early ELISA, Platelia™ NS1 and Dengue NS1 Ag Strip in the presence of IgM was 69.4%, 64.5% and 77.4%, respectively (Table 2). In this study, the presence or absence of IgM did not influence detection by the pan-E Early ELISA (*P* = 0,6159). However, a higher detection rate by both Platelia™ NS1 (Biorad) and Dengue NS1 Ag Strip (Biorad) in the absence of IgM was statistically significant (*P*<0,0001 and *P* = 0,0008, respectively).

### Sensitivities of the NS1 tests by the number of days after the onset of the symptoms

**Figure 1:**
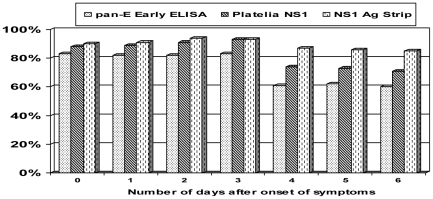
Sensitivity of 3 commercial dengue NS1 capture assays according to the number of days of illness (*n* = 220).

The sensitivities of all NS1 tests were evaluated according to the number of days of illness. A higher detection rate by the three tests analyzed was during the first four days after the onset of the symptoms (Day 3, in Figure 1, considering day 0 as the first day of fever). The sensitivity of Platelia™ NS1 (Biorad) decreased to 75% of detection after that and maintained the same rate until day 6 of illness. However, after the 4^th^ day, the NS1 Ag Strip (Biorad) showed 89.0% of sensitivity up to the 7^th^ of symptoms. From day five to the 7^th^, the pan-E Early ELISA (Panbio) confirms about 60.0% of the cases (Figure 1). Although dengue NS1 antigen detections up to the 9^th^ day are observed, here we plotted cases only up to the 7^th^ day due to the low number of samples representing 8^th^ and 9^th^ days in our population.

**Figure 2:**
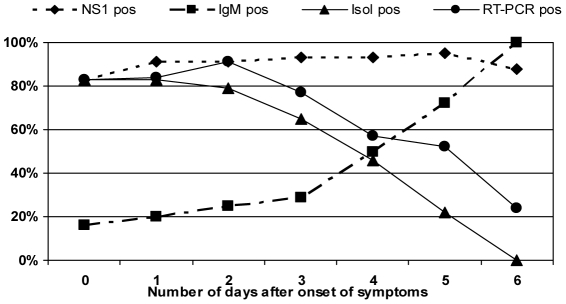
Overall sensitivity of positive results in any NS1 antigen capture assay compared to dengue diagnosis by MAC-ELISA, virus isolation and RT-PCR according to the number of days after onset of illness.

We also aimed to compare the cases confirmation by the dengue NS1 antigen capture to the confirmation by other methodologies used in this study according to the number of the days of illness. In this comparison, we considered a NS1 positive case, as a case positive in any of the three tests used. Figure 2 shows NS1 confirmation around 90% of the cases up to the 7^th^ day of illness, as previously shown. RT-PCR and virus isolation detections rate were around 80% in the first three days of illness, decreasing after that. However, on the other hand IgM detection rates increase only after the 4^th^ day of illness.

### NS1 sensitivity in primary and secondary infections

The serologic response could be characterized by IgG-ELISA in 54 samples, where a second specimen was available. There were 40 primary and 14 secondary infections. No differences were observed by the pan-E Early ELISA (Panbio) (*P* = 0.96) and by the NS1 Ag Strip (Biorad) (*P* = 0.76) in confirming primary and secondary infections (Table 3). However, the Platelia™ NS1 test showed a higher sensitivity in confirming primary infections than secondary ones (*P* = 0.01).

**Table 3:** Sensitivities of NS1 antigen capture assays in patients with primary and secondary dengue infections (*n* = 54).

	NS1 antigen capture kitsNo. of sera with indicated result/total no. tested^a^					
Patients immune response	pan-E DENGUE EARLY ELISA	*P* value	Platelia™ Dengue NS1 Ag-ELISA	*P* value	Dengue NS1 Ag STRIP	*P* value
Primary infection (*n* = 40)	26/40 (66.0)	0.96	38/40 (95.0)	0.01	38/40 (95.0)	0.76
Secondary infection (*n* = 14)	09/14 (64.3)		10/14 (71.4)		13/14 (93.0)	

a Values in parentheses are percentages.

### NS1 tests efficiency and predicted values

In our study, the pan-E Early ELISA test (Panbio) was less efficient in detecting acute dengue infections (86.1%) when compared to the Platelia™ NS1 test (91.3%) and the NS1 Ag Strip (95.0%). Positive predictive values were 98.3%, 99.5% and 100% for the Platelia™ NS1 (Biorad), NS1 Ag Strip (Biorad) and pan-E Early ELISA tests (Panbio), respectively. However, the pan-E Early ELISA (Panbio) showed the lowest negative predictive value (78.3%), followed by the Platelia™ NS1 test (Biorad) with 86.3% and the NS1 Ag Strip (Biorad) with 91.1% (Table 4).

**Table 4:** Sensitivity, specificity, efficiency and predictive values of 3 commercial dengue NS1 capture assays.

	NS1 antigen capture tests		
	pan-E Early ELISA (PanBio) (%)	Platelia NS1 (BioRad) (%)	NS1 Ag Strip (BioRad) (%)
Sensitivity	72	84	90
Specificity	100	99	99
Efficiency	86	91	95
Positive Predicted Value	100	98	99
Negative Predicted Value	78	86	91

## Discussion

The techniques of dengue serologic diagnosis which have been widely used are based on the detection of IgM antibodies by MAC-ELISA and IgG by IgG-ELISA. However, one of the limitations of these techniques is the inability to detect antibodies to DENV in the acute phase of disease [5], [26]. It takes 3 to 5 days for IgM antibodies and anti-DENV 10–14 days for IgG anti-DENV to become detectable. Moreover, primary and secondary infections have different profiles of production of these antibodies [27].

According to previous studies the presence of NS1 in human sera can be confirmed between days 0 to 9 [28], [29], [30] and with a peak at days 6 to 10 [31]. Currently, commercial kits such as the Dengue EARLY ELISA (Panbio Diagnostics, Brisbane, Australia), Platelia™ Dengue NS1 Ag-ELISA and Dengue NS1 Ag STRIP (BioRad Laboratories Marnes La Coquette, France) are available for early diagnosis of dengue based on NS1 antigen capture and several studies have been conducted in many laboratories [12], [15], [18], [19], [29], [31], [32], [33], [34], [35], [36].

In this study, we had the opportunity to evaluate and compare three NS1 antigen capture kits available with a panel of samples (*n* = 450) from cases occurred since the introduction of dengue in Rio de Janeiro, Brazil in 1986 to 2008. The NS1 Ag Strip test (Biorad) was the most sensitive in confirming dengue cases, followed by Platelia™ NS1 (BioRad). The least sensitive was the pan-E Early ELISA (PanBio) with 72.3% of sensitivity. However, in this study PanBio kit was the most specific (100%) while both kits from BioRad showed 98.7% and 99.1% of specificity, respectively. A recent evaluation in Malaysia showed that the NS1 Ag Strip had 90.4% of sensitivity and 99.5% of specificity [17]. Studies performed in Vietnam [18] and French Guyana [29] showed sensitivities of 82% and 88%, respectively for the Platelia™ NS1 test. However, sensitivities varying from 63.2% to 93.3% have also been reported for this kit [12], [15]. Even though different DENV genotypes may circulate in the Americas and Asia, NS1 kits evaluations in countries from those area show the ability of those tests to detect DENV in infected patients. Our observations are consistent with previous studies in which the pan-E Early ELISA had lower sensitivities [13], [14], [16], [37]. However, to increase diagnostic performance, Panbio has recently released an improved second generation for their NS1 capture kit with changes in key reagents and procedure [38].

All NS1 tests were more sensitive in confirming cases positive by virus isolation than in cases positive by RT-PCR. Dussart [29] confirmed 94.1% of cases positive by virus isolation and 85% of the cases RT-PCR positive using the Platelia™ NS1 test. Recently, McBride [16] showed that the NS1 antigen capture was positive in 87% of the cases positive by RT-PCR. In our study, the Dengue NS1 Ag Strip confirmed 98.7% of the cases positive by virus isolation and 82.3% of RT-PCR positive cases, results similarly observed by Zainah [17]. In the presence of IgM antibodies, the Dengue NS1 Ag Strip confirmed more cases (77.4%) than the pan-E Early ELISA (69.4%) and the Platelia™ NS1 (64.5%). The presence or absence of IgM did not influence in the cases confirmation by the pan-E Early ELISA (*P* = 0,6159). However, the higher confirmation by both Platelia™ NS1 and the Dengue NS1 Ag Strip in the absence of IgM were statistically significant. Sekaran [32] showed that the NS1 detection rates decrease as IgM levels rise, in agreement with our results.

The pan-E Early ELISA (PanBio) showed a higher sensitivity in confirming DENV-2 infections and the Platelia™ NS1 kit (BioRad) in DENV-1 infections. However, the Dengue NS1 Ag Strip kit (BioRad) showed the same sensitivity in confirming DENV-1 and DENV-2 infections. DENV-3 infections were the least confirmed by all three kits. The apparent inability in confirming infection by this serotype has been shown previously [33]. Furthermore, differences in the inter-serotype sensitivities have been reported for all three kits. McBride [16] recently showed lower sensitivities by the pan-E Early ELISA (PanBio) in DENV-2 and DENV-4 infections. The latter was also found in previous studies performed by Bessoff [37] and Dussart [14] and most recently in a study performed in Venezuela [36]. Due to the absence of DENV-4 circulating in Brazil, we were not able to access the assays sensitivities in cases infected by this serotype. Both Biorad kits (Platelia™ NS1 and Dengue NS1 Ag Strip) showed a lower sensitivity in DENV-2 infections from Vietnam [18] and Venezuela [36].

A higher detection rate by the three tests was found during the first four days after the onset of the symptoms. Although dengue NS1 antigen detections up to the 9^th^ day are described, here we analyzed cases only up to the 7^th^ day due to the low number of samples representing 8^th^ and 9^th^ days in our population. The lack of later samples in this study did not allow us to determine when NS1 detection would decrease. However, previous studies found NS1 antigen in 82% to 83% of patients with dengue from day 1 to 9^th^ after the onset of fever [11], [30].

The Platelia™ NS1 test showed a higher sensitivity in confirming primary infections than secondary ones, as previously observed [12], [15], [17], [18], [32], [34]. False negative results by NS1 antigen capture in secondary infections may also be due to the immune-complexes formation by the anti- DENV IgG sequestration [39]. Efforts to dissociate immune complexes by acid treatment can enhance the assays sensitivities, as previously shown [15]. However, in our study no attempts were made to dissociate those complexes. To further analyze the sensitivity of those tests in confirming secondary cases, a larger number of cases should be tested.

Among the kits evaluated, the Dengue NS1 Ag Strip (BioRad) was the most efficient in confirming dengue infections by capturing NS1 antigen from infected patients. Moreover, it was more convenient to be used, as the results can be obtained in 15 minutes, easy to perform and its performance does not involve the use of special laboratory equipment.

Previous studies have demonstrated a diagnostic strategy combining NS1 Ag detection in acute-phase sera and DENV IgM detection in early-convalescent-phase sera, providing a sensitivity of about 90% for dengue diagnosis [29], [34].

In conclusion, this evaluation has shown that NS1 antigen capture assays are indeed an alternative tool for the early diagnosis of dengue infections, may be used as a screening test prior virus isolation and used in combination with IgM capture can increase the rate of cases confirmation, especially in endemic areas where secondary infections are expected to occur due to the co-circulation of the different DENV serotypes, such as seen in Brazil.

This evaluation was performed for research purposes only and authors have no financial interest. The pan-E Early ELISA from Panbio and the Dengue NS1 Ag Strip from BioRad were kindly provided for evaluation.

## Supporting Information

Alternative Language Abstract S1 Translation of the abstract into Portuguese by Flavia Barreto dos Santos
(0.02 MB DOC)
