# Supplementary material for: Comparison of Three Commercially Available Dengue NS1 Antigen Capture Assays for Acute Diagnosis of Dengue in Brazil
Source: PLoS Negl Trop Dis. 2010 Jul 6;4(7):e738. doi: 10.1371/journal.pntd.0000738 (PMC2897844; doi:10.1371/journal.pntd.0000738)
Supplement: Alternative Language Abstract S1 — Translation of the abstract into Portuguese by Flavia Barreto dos Santos (0.02 MB DOC) [file pntd.0000738.s001.doc]

O dengue está associado com epidemias urbanas explosivas e se tornou um grande problema de saúde pública em muitos países em desenvolvimento, incluindo Brasil. O diagnóstico pode ser realizado utilizando diversas abordagens, porém métodos sensíveis e específicos úteis ao diagnóstico precoce são necessários. A proteína não-estrutural NS1 dos *Flavivirus*, uma glicoproteína altamente conservada e secretada, é uma candidata para o diagnóstico rápido do dengue em países endêmicos. O objetivo deste trabalho foi avaliar a potencial utilização de 3 kits comerciais de captura de antígeno NS1 com um painel de 450 amostras de soros para o diagnóstico precoce do dengue no Brasil. O kit PanBio Early ELISA (PanBio Diagnostics) apresentou uma sensibilidade de 72,3% (159/220) e uma especificidade de 100%, enquanto que a sensibilidade do kit Platelia™ NS1 (Biorad Laboratories) foi de 83,6% (184/220). Contudo, a maior sensibilidade (89,6%; 197/220) foi obtida utilizando o kit NS1 Ag Strip (Biorad Laboratories). Uma menor sensibilidade pelos 3 kits foi observada em casos de DENV-3. Soros positivos por isolamento viral foram mais frequentemente detectados do que casos positivos por RT-PCR e uma maior detecção foi observada durante os quarto primeiros dias após o início dos sintomas. A presença ou ausência de IgM mostrou não influenciar a confirmação pelo pan-E Early ELISA (*p=*0,6159). No entanto, uma maior confirmação dos casos na ausência de IgM pelo Platelia™ NS1 (Biorad) e pelo Dengue NS1 Ag Strip (Biorad) foi estatisticamente significativa (*p*<0,0001 e *p=*0,0008, respectivamente). Apenas o kit Platelia™ apresentou uma maior sensibilidade em confirmar casos de infecção primária. Os resultados indicam que os kits comerciais para a captura de antígeno NS1 são úteis para o diagnóstico de infecções primárias e secundárias agudas de dengue. Além disso, pode ser utilizado em combinação com o MAC-ELISA para a confirmação dos casos e como um método de triagem utilizado previamente ao isolamento viral.
